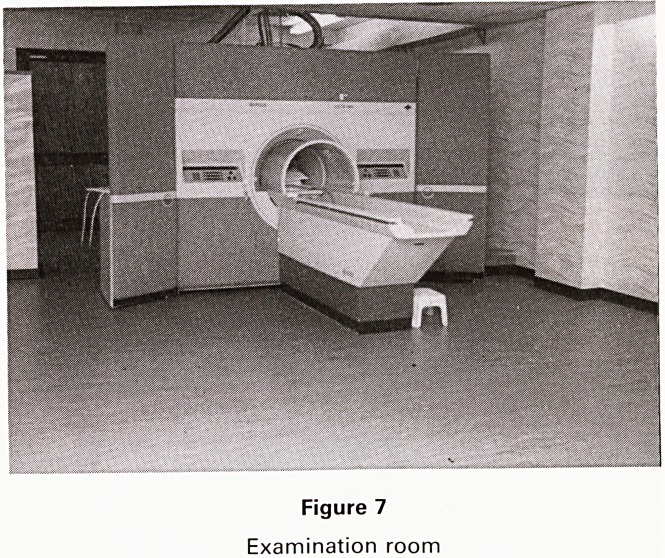# The Bristol MRI Centre

**Published:** 1988-05

**Authors:** Gordon Thomson


### The Bristol MRI Centre

**Gordon Thomson MD, FRCP, FRCR.**

One of the first requirements that followed the magnificent gift of a one million pound MRI Scanner by the John James Bristol Foundation to the three Bristol District Health Authorities, was a site for a building appropriate to the machine. Unfortunately no adequate space could be found in any of the existing hospital buildings, and approaches to the S.W. Regional Health Authority and all three D.H.A.s for a grant to help erect a new building proved fruitless, due to financial constraint. The Radiotherapy Dept. kindly offered a site on their roof, but access for ambulances would have been difficult! Southmead Hospital was already occupied with plans for the new Winford Hospital soon to be transferred to their campus.

Fortunately Frenchay Health Authority (F.H.A.) were able to offer three possible sites suitable for the purpose. One of these involved the demolition of several war-time Nissen huts, (Figures 1 and 2) but the potential of such a splendid offer, just beyond the existing swimming pool, and well away from the mainstream of hospital and patient activity, was immediately apparent, and a lease was arranged (not without some initial difficulties) at a peppercorn rent, for the three D.H.A.s to use the site, with right of equal access for their respective patients at all times. An unusual view was obtained following the demolition of the huts showing parts of the lovely grounds of the hospital with its fine trees (Figure III), with the chosen site in the foreground. We were very grateful to F.H.A. for allowing us to build on this site, and come to the agreed arrangement for the lease. The John James Foundation were also delighted to learn that space had been found in the grounds of an N.H.S. hospital, as this met the wish expressed at the time of the original donation.

Inasmuch as the N.H.S. was unfortunately unable to help fund the building, and in the initial phase furthermore the revenue costs as well, the challenge was made to go to public appeal for the money required. A suitable design for the building was chosen from three offered at a lunch-time meeting at the local Crest Hotel between representatives of Picker International (manufacturers of the MRI selected), the three radiologists concerned, Dr Terry Beddoe, and the architects. With impressive speed, the revised and completed drawings were submitted a few weeks later, and the contract to build on a turnkey basis was awarded to Cowlins, one of the major Bristol building contractors.

In the meantime, the Bristol MRI Scanner Fund was set up, and the Appeal launched in January 1986. The constitution included two patrons, Mr John James himself

**Figure 1**

War-time Nissen huts on the site offered by F.H.A.

**Figure 3**

The site cleared

**Figure 2**

Demolition in progress

**Figure 4**

The completed MRI Centre and Lady Middlemiss together with four Trustees, responsible for organising the development, and raising the necessary finance. Dr Terry Beddoe, a retired G.P. and ex-medical officer to British Aerospace, became Chairman of the Trustees; Paul Goddard represented the Royal Infirmary, Clive Johnson Southmead Hospital and Gordon Thomson Frenchay Hospital. The building was due to cost over half a million pounds, and the first year's running costs were to be the total responsibility of the Trust (about £100,000). In year two it was agreed that the three D.H.A.s would contribute 25% of the costs, in year three 50%, year four 75% and in year five the total running costs, with ownership thereafter by the N.H.S. As the service charge (£65,000) had to be added to the total costs in year two and after, annual revenue costs of about £200,000 therefore had to be visualised. The task for the Trustees indeed looked pretty formidable!

However, the public response was superb, with a total of over £700,000 raised within two years. We would like to express our deep and sincere gratitude to the many, many people who helped to raise this considerable amount so quickly. To the pubs and clubs, the walkers, the runners, parachutists, balloonists and swimmers, and to the general public at large, who in so many different ways, both in Bristol and outside, rallied round and supported us. In particular, we would like to thank Frank Ross and Hugh Coakham who were part of the fund-raising team, and the Dr Jazz Group, who under the inspiring leadership of Paul Goddard, produced many hours of stimulating music. The Trustees have done their best to send a thank-you letter for every donation, but if anyone has inadvertently been left out, we make our apologies. To all concerned therefore, a sincere thank you for your support.

The splendid building was finished (Figure IV) two weeks ahead of schedule, to everyone's immense pleasure and satisfaction. The interior design and furnishings (Figures 5, 6, 7) were of a high standard, and provoked numerous admiring comments from patients and visitors alike. The whole atmosphere has proved excellent for staff working relationships, whilst at the same time promoting confidence and relaxation in patients who often arrive apprehensive and concerned about the unknown technology. The fact that the Centre is entirely self-contained and remote from the main hospital complex has also proved beneficial for happy relationships.

The staff were appointed well ahead of the opening date, and consisted of two full-time radiographers and two part-time secretary-receptionists. Ann Case from the Hammersmith MRI Unit was appointed radiographer in charge, and supported by Joanne Waring from Cambridge's Addenbrooke's Hospital. Gloria Hatton and Rona Wright became the secretary-receptionists. The day to day and long term running of the Centre became the responsibility of the Trustees, working in an honorary capacity. Patient examination commenced early in June 1987 and within the first year about 1600 patients have been examined. The week has been divided equally between the three D.H.A.s, each one having three sessions of time on the machine. The tenth one is reserved for maintenance procedures by the Picker engineer. The staff employed by the Centre remains the same throughout the week, but radiologists from the user hospital attend at the time their patients are examined. A Monday lunch-time meeting has been a regular feature for radiologists to attend whilst the previous week's films are reviewed. This has allowed general experience to be gained, as each hospital produces a medical input distinct from the other two. A considerable library of typical and unusual cases has thus been quickly assembled.

**Figure 5**

Reception area

**Figure 6**

Patient waiting area

**Figure 7**

Examination room

The Centre was officially opened on June 29th 1987 by John James, C.B.E., LL.D., a speech of appreciation was made by Geoffrey Mortimer, Chairman of Frenchay Health Authority, and a splendid reception followed at District Headquarters. The citizens of Bristol in particular give thanks for his magnificent gift. Many patients will benefit from his generosity.

## Figures and Tables

**Figure 1 f1:**
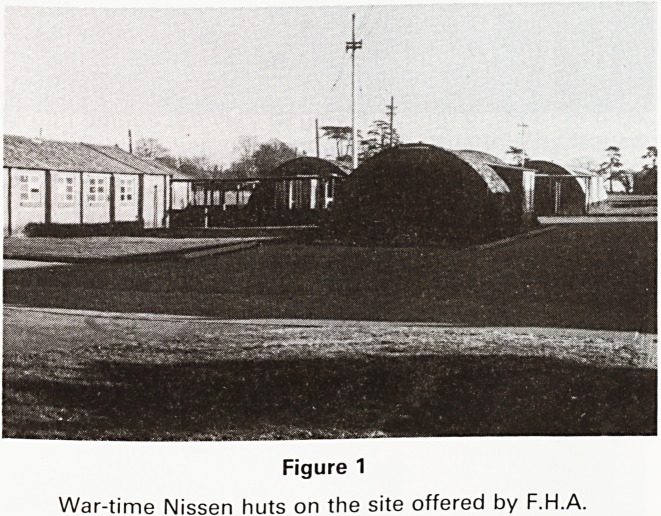


**Figure 2 f2:**
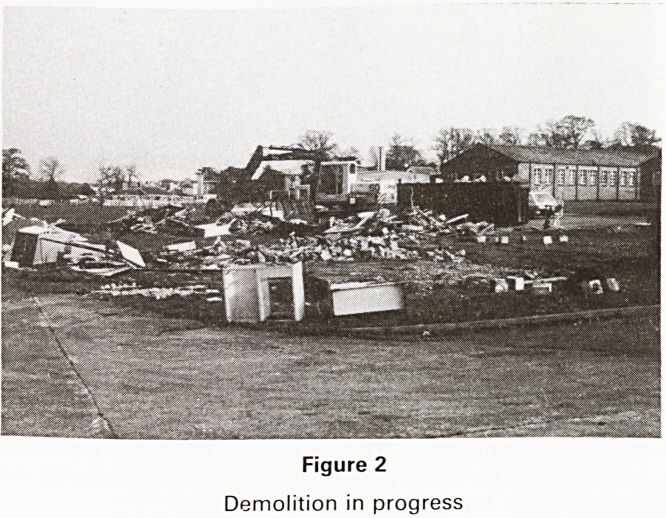


**Figure 3 f3:**
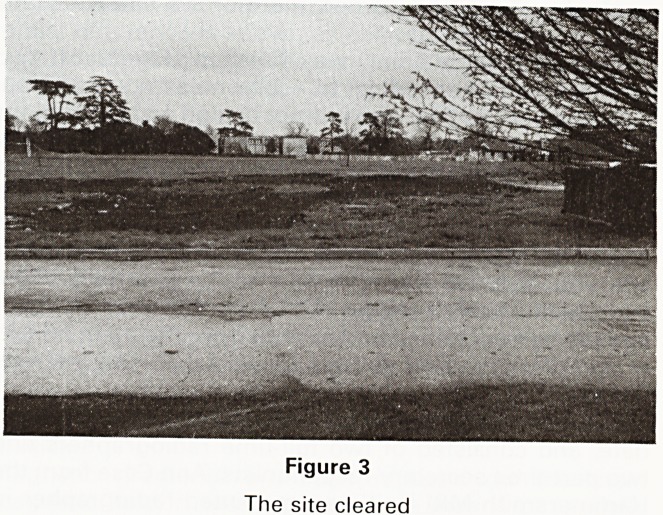


**Figure 4 f4:**
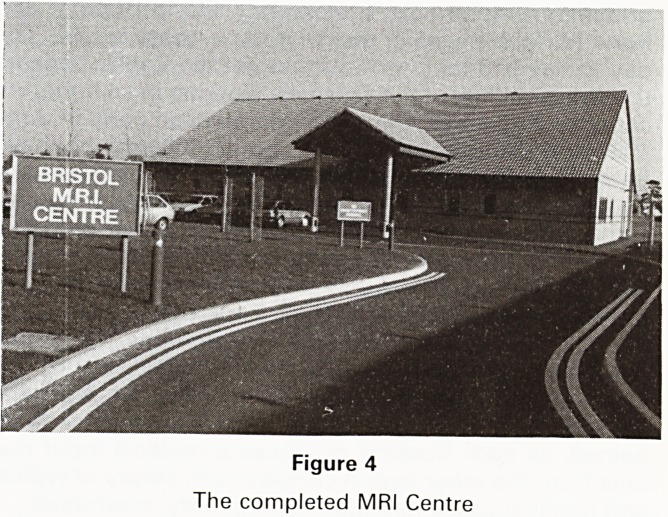


**Figure 5 f5:**
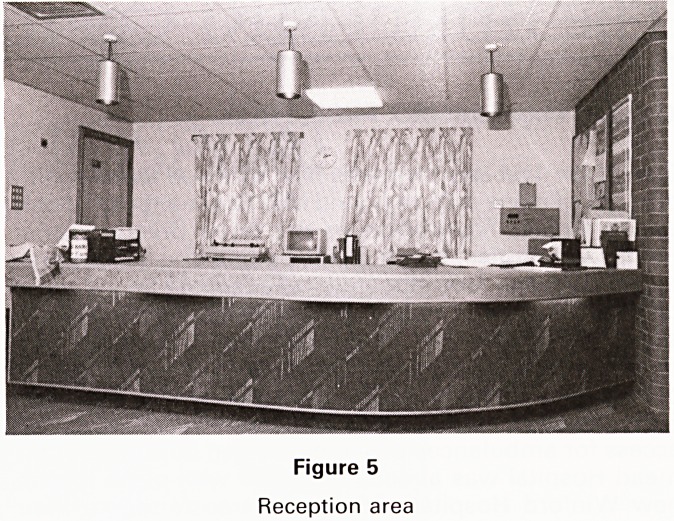


**Figure 6 f6:**
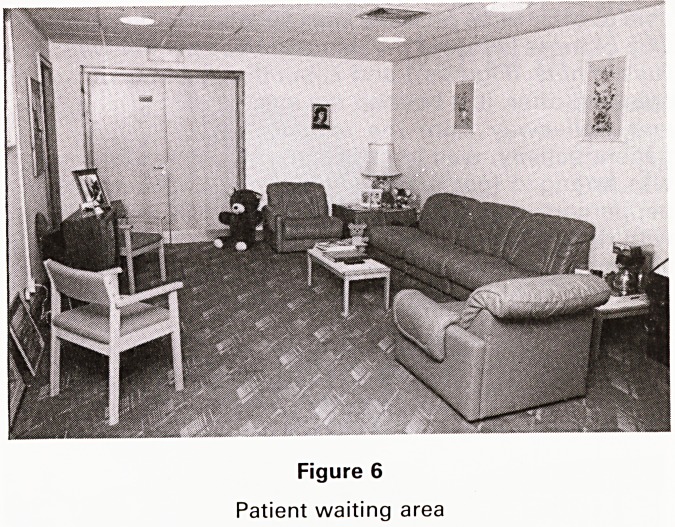


**Figure 7 f7:**